# Clinical and Immunological Efficacy of Aspirin Desensitization in Nasal Polyp Patients with Aspirin-Exacerbated Respiratory Disease

**Published:** 2017

**Authors:** Negar Mortazavi, Hossein Esmaeilzadeh, Mohammad Abbasinazari, Delara Babaie, Soheila Alyasin, Hesamodin Nabavizadeh, Elmira Esmailzadeh

**Affiliations:** a *Department of Clinical Pharmacy, School of Pharmacy, Shahid Beheshti University of Medical Sciences, Tehran, Iran. *; b *Allergy Research Center, Shiraz University of Medical Sciences, Shiraz, Iran. *; c *Department of Allergy and Clinical Immunology, Mofid Hospital, Shahid Beheshti University of Medical Sciences, Tehran, Iran.*; d *Department of Internal Medicine, Division of Rheumatology, Shiraz University of Medical Sciences, Shiraz, Iran.*

**Keywords:** AERD, Aspirin, Desensitization, Interleukin-5, Interleukin-4

## Abstract

This study aimed to investigate the efficacy and the underlining mechanism of aspirin desensitization among patients with Aspirin Exacerbated Respiratory Disease (AERD). Thirty-eight patients, who had undergone an aspirin challenge test and were diagnosed as having AERD, were engaged in a double-blind randomized clinical trial. They were divided into two groups—an active group of patients who went through aspirin desensitization, and the control group, receiving placebo. Clinical symptoms and the quality of life of the patients—in addition to the levels of interleukin 4 and 5 (IL4), (IL5)—were documented at the beginning of the study and again after six months of aspirin desensitization. The quality of life of the patients was significantly higher in the active group after six months (*P* = 0.001). Medication requirements and symptom score were manifested to be significantly lower in the active group after six months than at the beginning of the study (*P* = 0.005, 0.017 respectively). Forced expiratory volume in the second one (FEV1) was, also, significantly higher in the active group after six months of the study (*P* = 0.032). IL5 was found to be significantly lower in the active group after six months (*P* = 0.019). However, no significant difference was observed in the levels of IL4 between the two groups (*P* = 0.152). The study revealed that aspirin desensitization can improve the quality of life of patients with AERD, lessen their symptoms and medication requirements, lower their levels of IL5, and improve some pulmonary function tests such as FEV1.

## Introduction

Nonsteroidal anti-inflammatory drugs (NSAID) are widely used for treating inflammatory disease. However, their use may be restricted due to the hypersensitive reactions varying from mild skin or airway symptoms to severe systemic manifestations and anaphylactic shock (1). The reactions to the NSAID are classified into IgE and non-IgE mediated ones. The Aspirin Exacerbated Respiratory Disease (AERD), also known as the NSAID-Exacerbated Respiratory Disease (NERD), is the most prevalent non-IgE mediated reaction, which is accompanied by asthma exacerbation, respiratory distress, or nasal symptoms (2). The prevalence of the AERD is not only higher among the patients with chronic rhinosinusitis with nasal polyposis (CRSwNP) and/or asthma, but also more severe among these patients (3).

Nasal polyposis is a fibroblastic tissue defined by chronic inflammation of the upper airways. Several factors are suggested to be involved in its pathophysiology, including aspirin intolerance, chronic infection, inhalant, and/or food allergy (4). One study detected that aspirin intolerance can increase the release of eosinophil-related mediators in nasal polyposis (5). Another survey manifested that aspirin hypersensitivity can also play a role in the persistence and severity of nasal polyposis by inhibiting the apoptosis of inflammatory cells in this disease (5). 

Several hypotheses were suggested regarding the aetiology of aspirin intolerance. Some studies insisted on the role of genetic polymorphism in drug hypersensitivity including aspirin intolerance (6). Other surveys have reported the role of HLA-DQB1*0301 and HLA-DRB1*011 in the pathogenesis of the AERD (7).

In addition to nasal polyposis, it was also declared that aspirin intolerance may also be involved in aspirin-induced asthma by deregulating Cyclooxygenase-2 (COX-2), which is the main inflammatory mediator in protecting prostaglandin E2 (PGE2), a protective mediator in the AERD (8). Considering the relationship between nasal polyposis and asthma with aspirin hypersensitivity, several studies were conducted to prove the efficacy of aspirin desensitization as an effective treatment for aspirin intolerance. Waldram *et al.* introduced aspirin desensitization by daily aspirin therapy as an effective treatment for the AERD (9). Aspirin desensitization was also introduced as an effective treatment for improving the sense of smell and reducing the need for surgery to avoid the recurrence of nasal polyps in the AERD (10, 11). 

In regards to the efficacy of aspirin desensitization in the AERD, some clinical trials concluded that aspirin desensitization can improve asthma control and symptom score as well as decrease the medication need among the AERD-affected patients (12-14). In addition to evaluating the clinical improvement in the AERD by aspirin desensitization, some studies focus on the effect of this method on immunologic profiles. In their study, Aksu *et al.* detected that aspirin desensitization can cause declines in the levels of CD4 (+) IFN-gamma and CD4 (+) IL-10, and can also reduce these to normal values after one month of desensitization. However, this method had no effect on the level of lipoxin (15). Another survey, conducted on the effect of aspirin desensitization on inflammatory biomarkers involving in the AERD, detected that this method can reduce IL-4 and matrix metalloproteinase 9 (MMP9) after six months of therapy (16). Some other studies also concluded that performing aspirin desensitization by inhibiting the inflammatory pathway leading to IL-4 and IL-13 production can be effective in chronic rhinosinusitis with nasal polyposis (CRSwNP) (17).

Considering gene variability in the pathogenesis of aspirin hypersensitivity as well as the different effects of aspirin desensitization on aspirin intolerance, especially on the biochemical markers of the disease, it seems to be essential to perform clinical trials on different sections of the population with various ethnicities. The aim of this study is to investigate the efficacy and probable mechanism of aspirin desensitization among Iranian patients. 

## Experimental

This randomized clinical trial was conducted in two referral centres of immunology and allergy, affiliated to the Shiraz University of Medical Sciences in South Iran and the Shahid Beheshti University of Medical Sciences in Central Iran, from May 2015 to January 2017.

The clinical trial was registered in IRCT as IRCT2016070728821N1 and the ethical committee’s approval was obtained from the Shahid Beheshti University of Medical Sciences as per the provisions of Helsinki (18). 

*Selecting the patients and criteria for inclusion*

As many as 38 AERD patients were completed and analysed in this survey. Their age varied from 19 to 54 years. The diagnosis of AERD was based on the aspirin challenge test using a method propounded by Esmaeilzadeh *et al.* (19). The aspirin was administered in increasing dosages of 20, 40, 80, and 325 mg at intervals of 90 min. Forced expiratory volume in the second one (FEV1) was measured before and after 30 min of administering each dosage of aspirin.

If the decrease in FEV1 was more than 15% and accompanied by extra-bronchial symptoms such as nasal congestion, rhinorrhea, or urticaria during the test, the patient would be considered to have AERD, and was included in the survey. 

*Exclusion criteria*

Patients with a history of anaphylactic or type-1 hypersensitivity reaction to aspirin, a history of gastrointestinal bleeding or bleeding incontinence, liver dysfunction, the ones who were not willing to enter the study, and pregnant women were excluded from this trial. 

*Aspirin desensitization method in selected patients*

This study is a double-blind placebo control clinical trial (RDBCT). The selected patients were divided into two groups—active and placebo—through a random computer-generated division in block size. The active group went through aspirin desensitization and the control group received placebo. In order to keep the participating clinicians blind to the patients’ allocation, the aspirin and placebo capsules were prepared by a pharmacy student, who was not engaged in visiting the patients.

By using 80 mg and 325 mg tablets of aspirin and a pill cutter, capsules with different dosages of aspirin were provided. Similar capsules containing starch were administered to the control group. Besides study intervention, participants were allowed to take standard medications to control their asthma or nasal symptoms.

For aspirin desensitization, a two-day method was applied, starting with intranasal ketorolac spray in two-fold increasing dosage at an interval of 30 min, followed by two dosages of 60-mg aspirin/placebo at regular intervals of 90 min on the first day (20). On the second day, the aspirin/placebo was administered in dosages of 160 and 325 mg at an interval of 180 min. Afterwards, for the maintenance therapy, patients received 650 mg of aspirin/placebo twice every day for one month and 325 mg of the same twice daily for the next five months.

Six months after initiating the aspirin/placebo dosages, asthma attacks, recurrence of nasal polyposis, FEV1, symptom score (19), and medication need score (19) were determined and recorded for each patient. For the sinonasal quality of life, the patients were assessed through a sinonasal outcome test (SNOT-22) during each visit (21). Lower scores of SNOT-22 indicate a higher quality of life among the patients.

After six months, a CT scan was done and the nasal polyp Lund-MacKay score of each patient was identified (22). The levels of interleukin 4 and 5 (IL4 and 5) were also measured before initiating the study and also at its end. 

*Statistical Analysis*

Data were entered in the Statistical Package for Social Sciences, Version 19 (SPSS 19). The Mann–Whitney U test was applied in order to compare the continuous data and the Chi-Square test was used to analyse the categorized variables. The significant *p*-value was considered to be less than 0.05 in all these comparisons. 

## Results

**Figure 1.**
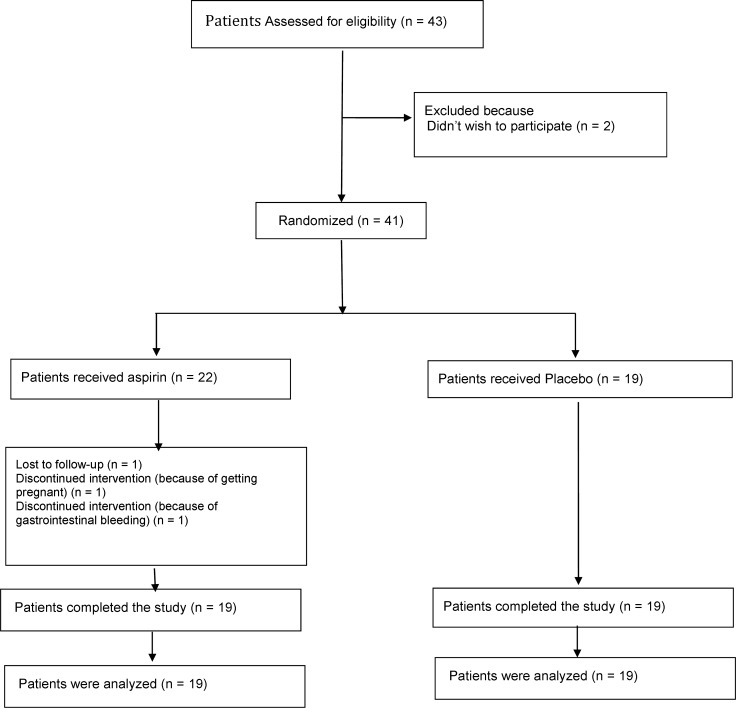
The number of patients entered in the study and remained up to end of the study

The mean age of the patients in the active group was about 33 ± 2 years old and the mean age of the ones in the control group was about 29 ± 1 years old. The number of patients who participated in the study and remained until the end of the study was shown in Figure 1.

**Figure 2:**
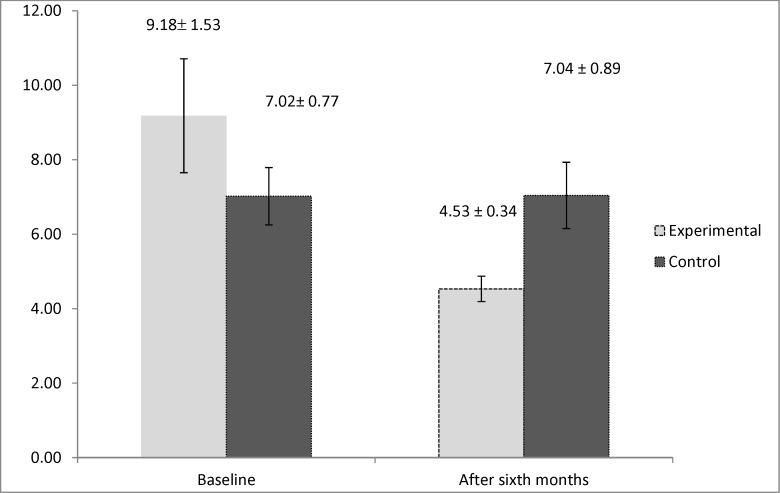
Interleukin 5 (IL5) mean values in experimental group (underwent aspirin desensitization) and control group (receiving placebo

**Figure 3:**
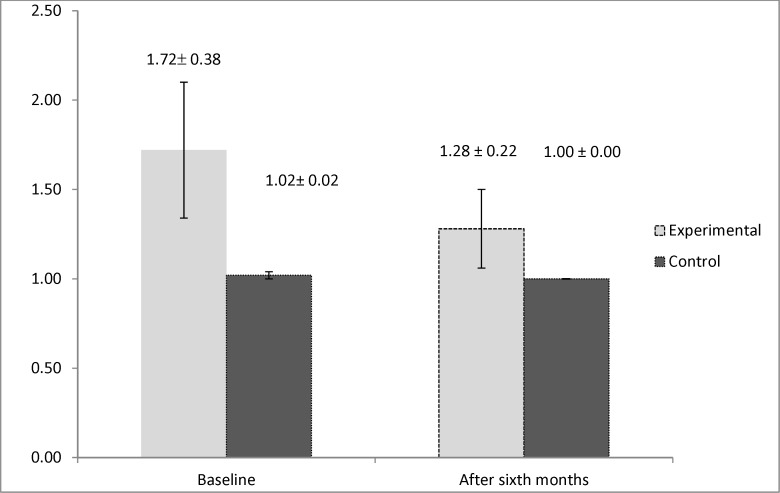
Interleukin 4(IL4) mean values in experimental group (underwent aspirin desensitization) and control group (receiving placebo

**Table 1.** Clinical outcome assessment between experimental group (underwent desensitization) and control group (receiving placebo

**Clinical Assessment **	**Experimental Group (Mean ± SD)**	**Control Group (Mean ± SD)**	***P*** **-value**
SNOT (0)	53.95 ± 14.4	41.53 ± 9.9	0.011
SNOT (1)	42.74 ± 14.7	38.42 ± 9.5	0.248
SNOT (6)	27 ± 10.8	39.63 ± 10.5	0.001
Lund-MacKay(0)	14.74 ± 3	12.42 ± 1.1	0.014
Lund-MacKay(6)	11.26 ± 2	12.05 ± 19	0.229
Symptom Score (0)	15.37 ± 4.8	12.42 ± 3.9	0.063
Symptom Score (1)	12.53 ± 4.06	11.95 ± 3.5	0.49
Symptom Score (6)	7.95 ± 3.4	11.89 ± 4.2	0.005
Medication Score (0)	13.05 ± 1.3	11.47 ± 2.2	0.021
Medication Score (1)	12.74 ± 1.5	11.58 ± 2.6	0.274
Medication Score (6)	9.47 ± 1.8	11.47 ± 2.7	0.017

(0): at the beginning of the study.

(1): 1 month after initiating the study.

(6): 6 months after initiating the study.

**Table 2:** Asthma severity assessment

**Asthma Assessment**	**Experimental Group (Mean ± SD)**	**Control Group (Mean ± SD)**	***P*** **-value**
FEV1 (0)	74.68 ± 5.9	81.84 ± 7.8	0.005
FEV1 (1)	78.53 ± 7.1	78.53 ± 8	0.918
FEV1 (6)	85.37 ± 5.9	80 ± 7	0.032

(0): at the beginning of the study.

(1): 1 month after initiating the study.

(6): 6 months after initiating the study.

There was no significant difference between these two values in the groups mentioned. Besides, the gender distribution was also almost the same in both the active and the control groups with 10 males and nine females.

The assessment of the clinical outcome of the patients showed that the SNOT 22 score was significantly higher in the active group at the beginning of the study (*P* = 0.011); however, after six months, this score was significantly higher in the control group (*P* = 0.001). In addition, the Lund-MacKay score was significantly higher in the active group at the beginning of the study (*P* = 0.014) and no significant difference was observed after six months. The symptom score was also demonstrated to be significantly higher in the control group compared to the active group after six months of the study (*P* = 0.005). Besides, the medication need score was also found to be significantly higher in the active group at the beginning of the survey (*P* = 0.021); however, it had no significant difference with the control group after one month of the study and increased significantly in the control group after six months of the study (respectively *P* = 0.274, 0.017). The mean value of each clinical criterion is shown in Table 1.

The results on asthma severity and attacks showed significantly higher FEV1 among the patients in the control group at the beginning of the study (*P* = 0.005). However, this decreased significantly in the control group after six months (*P* = 0.032). The mean value of FEV1 in both the groups is summarized in Table 2. Moreover, the result also manifested that 36.8% (14 out of 38) of the patients had at least one episode of asthma attack. Among these 14 patients, nine were among those who received placebo and five went through aspirin desensitization. 

However, no significant difference was found in the rate of asthma attack between these two groups.

By evaluating the immunologic markers, we observed higher levels of interleukin 5 in the control group after six months of the study (*P* = 0.019). However, the result for the study of the levels of interleukin 4 demonstrated that it had significantly higher values in the active group at the beginning of the study (*P* = 0.033). This marker had no significant difference between two groups after six months (*P* = 0.152). The mean values of the immunologic markers are shown in Figures 2 and 3.

## Discussion

Numerous studies have been conducted on the clinical efficacy of aspirin desensitization in the nasal polyp; however, most of them were open trials (11, 12). Besides these, a number of recent double-blind clinical trials also manifested the clinical efficacy of aspirin desensitization in nasal polyp affected by the AERD (19). However, the mechanism of aspirin desensitization efficacy is still obscure. Our study, as a double-centre RDBCT, was focused on the clinical and immunological mechanism of aspirin desensitization among Iranian patients.

The results of evaluating the clinical outcome of aspirin desensitization in AERD patients demonstrated that the SNOT 22 score was significantly higher in the active group at the beginning of the study. This indicates towards a lower quality of life among these patients, which was not deliberate due to the random sampling used in the survey. Despite that, after six months of the study, the score got significantly lower in the active group compared to its counterpart. This means that aspirin desensitization can improve the quality of life of the patients in six months of therapy. A study conducted by Cho *et al.* also concluded that post endoscopic sinus surgery aspirin desensitization could reduce the SNOT22 score among patients with AERD (23). Another survey conducted by Havel *et al.* also manifested that aspirin desensitization can improve the quality of life scores as well as the sinusoidal symptoms among CRSwNP-affected patients (24). 

Besides, in order to assess nasal polyp objectively, a CT scan of the sinus was performed at the beginning and at the end of the study. After six months, according to the Lund-Mackay score of the CT scan, no significant difference was detected among the patients in the active and the control groups. However, due to the higher scores in the active group at the beginning of the study, it can be claimed that after six months of aspirin desensitization, the severity of sinus involvement in the active group decreased almost as much as the value in the control group. Some other studies also concluded that aspirin desensitization could also be an effective treatment method for the chronic inflammatory process involved in chronic rhinosinusitis (17, 25). Aspirin desensitization was also revealed to be a safe and effective treatment method for chronic hyperplastic sinusitis and nasal polyposis (26). 

The symptom score and the medication score were both observed to be significantly lower in the active group after six months of the study. This means better disease control, as well as lower medication, need after six months of aspirin desensitization. This result is compatible with the previous studies that concluded that aspirin desensitization can improve symptoms of the disease and also decrease the medication need among patients affected by the AERD (11, 13 and 19). 

The result of asthma severity showed that the FEV1 had higher values in the active group after six months of aspirin desensitization, which suggests better asthma control in accordance with spirometry results. Nevertheless, there was no significant difference in the number of asthma attacks among the patients of the active group and the control group. It suggests that although aspirin desensitization can improve the FEV1 result in cases of aspirin-sensitive asthma, it cannot reduce the number of asthma attacks. Some other surveys supported our result on asthma severity, however, most of them did not directly assess the effect of this treatment on the number of asthma attacks. For example, a clinical trial in Poland concluded that aspirin desensitization could improve clinically aspirin-induced asthma by boosting the pulmonary function test parameters (27). Moreover, another study—assessing the long-term effects of aspirin desensitization on uncontrolled asthma—manifested that it can improve FEV1 and the level of asthma control in the uncontrolled group (28). A long-term study on the efficacy of aspirin desensitization among asthmatic patients with AERD revealed that aspirin desensitization for at least six months could be effective in controlling asthma in AERD-affected patients for up to five years (16). Our results also showed that aspirin desensitization could be efficacious for asthma patients with AERD after six months of therapy. On the other hand, Nasser *et al.* declared that after acute aspirin desensitization in aspirin-sensitive asthma, urinary leukotriene 4 was still being produced and no change in the pulmonary function was observed after the desensitization (29). It also emphasized the importance of maintaining desensitization for at least six months in order to improve aspirin-induced asthma.

One of the main cells found in the nasal polyp tissue pathology is eosinophil, which is also implicated in the pathogenesis of AERD (5). Two main cytokines having influence in the growth and survival of eosinophil are IL4 and IL5 (30, 31). Furthermore, evaluating the effect of aspirin desensitization on these two immunologic markers manifested that aspirin desensitization can also reduce the amount of interleukin 5 significantly after six months of therapy. However, the amount of interleukin 4 had no significant change after six months of aspirin desensitization. The study conducted by Aktas *et al.* also declared that aspirin desensitization had no beneficial effect on interleukin 4 in AERD-affected patients (32). However, according to a review article, aspirin desensitization by inhibiting the production of interleukin 4 can also be effective on AERD (33). No specific study could be found that assessed the effect of aspirin desensitization on the levels of interleukin 5, hence, our study can be named as the first survey investigating this immunologic marker in AERD. Other surveys focused on interleukin 10 and other immunologic markers involved in the pathogenesis of AERD. According to one study, aspirin desensitization was also shown to have no effect on reducing IL 10, IFN-γ, and TGF-B (19). This controversy found in previous surveys can be attributed to genetic variability in response to aspirin desensitization (34, 35).

Two main limitations of our study were the number of patients and its length, which may be better if it is extended to one year or more. However, due to low compliance among patients, this could not be put into action. 

In spite of some limitations that was indispensable in a clinical trial, our study can be introduced as a distinct survey conducted on aspirin desensitization and its effect on AERD and aspirin-induced asthma in a significant number of patients compared to previous studies. Besides, our results on the positive effects of aspirin desensitization on interleukin 5 can be named as the first survey done on this immunologic factor. However, more multicentre studies are needed to evaluate the effects of aspirin desensitization on immunologic markers.
